# Central nervous system pathology in preclinical MPS IIIB dogs reveals progressive changes in clinically relevant brain regions

**DOI:** 10.1038/s41598-020-77032-y

**Published:** 2020-11-23

**Authors:** Martin T. Egeland, Marta M. Tarczyluk-Wells, Melissa M. Asmar, Evan G. Adintori, Roger Lawrence, Elizabeth M. Snella, Jackie K. Jens, Brett E. Crawford, Jill C. M. Wait, Emma McCullagh, Jason Pinkstaff, Jonathan D. Cooper, N. Matthew Ellinwood

**Affiliations:** 1 The Lundquist Institute at Harbor-UCLA Medical Center, and David Geffen School of Medicine, UCLA, Torrance, CA USA; 2 King’s College London, Institute of Psychiatry, Psychology and Neuroscience, Maurice Wohl Clinical Neuroscience Institute, London, UK; 3 BioMarin Pharmaceutical Inc., Novato, CA USA; 4 Iowa State University, Ames, IA USA; 5 Present Address: Department of Pediatrics, Washington University School of Medicine in St Louis, 660 S Euclid Avenue, Campus, Box 8208, St Louis, MO 63110 USA

**Keywords:** Lipid-storage diseases, Neurodegeneration

## Abstract

Mucopolysaccharidosis type IIIB (MPS IIIB; Sanfilippo syndrome B) is an autosomal recessive lysosomal storage disorder caused by the deficiency of alpha-*N*-acetylglucosaminidase activity, leading to increased levels of nondegraded heparan sulfate (HS). A mouse model has been useful to evaluate novel treatments for MPS IIIB, but has limitations. In this study, we evaluated the naturally occurring canine model of MPS IIIB for the onset and progression of biochemical and neuropathological changes during the preclinical stages (onset approximately 24–30 months of age) of canine MPS IIIB disease. Even by 1 month of age, MPS IIIB dogs had elevated HS levels in brain and cerebrospinal fluid. Analysis of histopathology of several disease-relevant regions of the forebrain demonstrated progressive lysosomal storage and microglial activation despite a lack of cerebrocortical atrophy in the oldest animals studied. More pronounced histopathology changes were detected in the cerebellum, where progressive lysosomal storage, astrocytosis and microglial activation were observed. Microglial activation was particularly prominent in cerebellar white matter and within the deep cerebellar nuclei, where neuron loss also occurred. The findings in this study will form the basis of future assessments of therapeutic efficacy in this large animal disease model.

## Introduction

Mucopolysaccharidosis type III (MPS III—Sanfilippo syndrome) is a group of autosomal recessive lysosomal storage disorders (LSDs), each caused by a specific enzymatic deficiency in the catabolic pathway of the glycosaminoglycan (GAG), heparan sulfate (HS)^1^. GAGs including HS are normally degraded by step-wise enzymatic modification removal of terminal sugars and sulfates at the non-reducing ends of GAG chains^2^.Therefore, individual deficiency in the enzymes involved in these catabolic pathways results in the lysosomal storage of non-degraded GAGs. Mucopolysaccharidosis type III is a heterogeneous condition comprised of four disorders in humans, MPS IIIA–IIID (OMIM 252900, 252920, 252930, and 252940). Each MPS III subtype is characterized by mutations in one of four distinct enzymes, each with an obligate and non-redundant role in HS catabolism. Deficiencies in the activities of each of these enzymes result in the accumulation of a disease-specific HS moiety at the non-reducing end (HS-NRE)^2^. Unlike most MPS disorders, MPS III patients have severe central nervous system (CNS) symptoms with relatively few peripheral disease manifestations.

MPS IIIB accounts for approximately 30% of all MPS III cases and is caused by mutations in the *NAGLU* gene, which encodes the enzyme alpha-*N*-acetylglucosaminidase (NAGLU*;* EC 3.2.1.50)^3–5^. Patients appear normal at birth but present with early developmental delays followed by severe behavioural abnormalities and progressive neurodegeneration. Patients typically succumb to their disease in their mid-teens^1^.

There is currently no approved treatment for MPS IIIB that targets the underlying disease. The challenge of developing effective therapies can be aided by the characterization of animal models that recapitulate important aspects of the human disease. A NAGLU deficient mouse (MPS IIIB mouse) model has been generated^6^, which display increased levels of HS and the MPS IIIB-specific HS-NRE in brain tissue^7, 8^, and neuropathological findings similar to those described in the human disease, including increased microgliosis and astrocytosis in the brain^9^. Treatment of MPS IIIB mice with recombinant human NAGLU (rhNAGLU) or AAV9-murine *Naglu* resolved biochemical, pathological and behavioural manifestations of murine MPS IIIB^7,10,11^ .

Despite such advances in MPS IIIB mice, small animal models are limited by a relative lack of neurological complexity and differences in physiological dynamics, making assessments of potential treatments for human use difficult. Larger animal models can provide additional, complementary disease pathology information and are well-suited for confirming the safety and pharmacologic activity of potential therapeutics before initiation of human testing. These models better represent the human disease and response to therapy because of the greater genetic similarity to humans, outbred status, robust immune system, greater size (including brain size and complexity), and longer lifespan. Large animal models of the MPS disorders have long been a productive focus of preclinical research in these disorders^12^. Specifically, large animal models of MPS IIIB have been described in emus^13^, cattle^14^, swine^15^, and canines, but only the canine model exists in a research setting suitable to evaluate a preclinical therapy^16^. A canine model of MPS IIIB has been described in the schipperke breed. Two dogs with naturally occurring MPS IIIB were found to have physical signs of and adult onset at approximately three years of age, of cerebellar disease including ataxia and tremor^17^. Gross pathological analysis of these dogs at 4.2 and 5.6 years of age revealed atrophy of the cerebellum with Purkinje cell loss, neuronal cytoplasmic vacuolation/granulation, and microglial vacuolation throughout the CNS, with somatic disease restricted chiefly to liver, kidney and tissue resident macrophages throughout the body^17^. The molecular defect in the canine *NAGLU* locus is involves the insertion of approximately 45 A residues flanked by an 11 base pair duplication of native sequence 5′ to the insertion^16^ in the last exon of the gene. Hence, while the initial clinical and molecular characterization has occurred, a longitudinal biochemical and histopathological assessment of the CNS disease in the current outbred colony of MPS IIIB dogs has not yet been conducted.

In this study, we describe the onset and progression of biochemical and neuropathological changes in the CNS of MPS IIIB dogs and unaffected control dogs at different ages during development up to 20 months of age, before the time point when these dogs develop clear motor signs. The biochemical characterization of the kinetics of substrate accumulation focused on the cortical grey matter and the cerebrospinal fluid (CSF). Histopathological analyses centred on the cerebellum as a known area of disease pathology. Other brain regions associated with the symptomatology of human MPS IIIB were also examined including the hippocampus, thalamus, and hypothalamus. In addition to obtaining baseline natural history data to document progressive biochemical and neuropathological changes in preclinical MPS IIIB dogs, our goal was to determine measures that could inform therapeutic benefit of potential future treatments for MPS IIIB disease.

## Results

**Figure 1:**
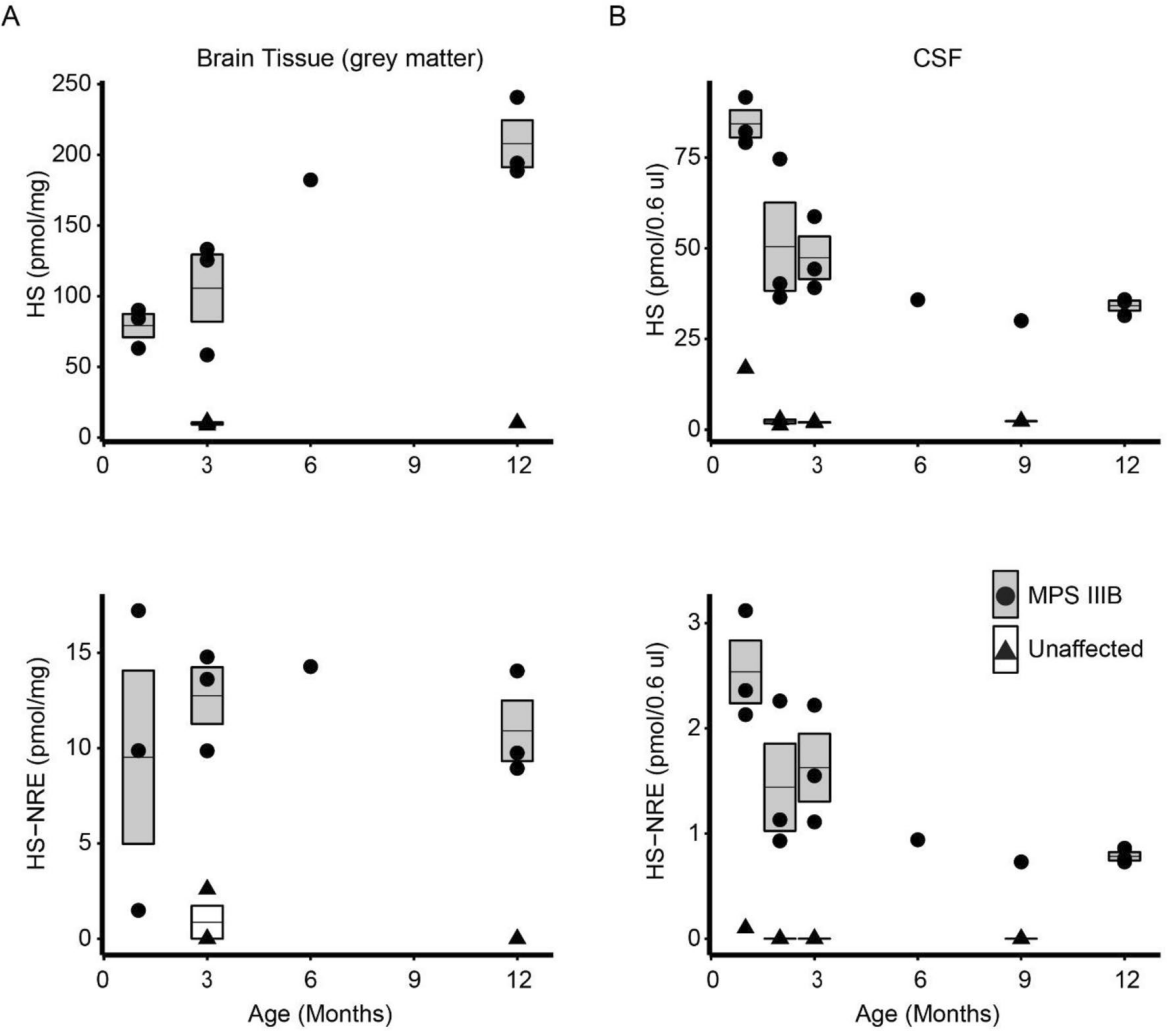
Increased levels of heparan sulfate (HS) and the MPS IIIB-specific non-reducing end of HS (HS-NRE) in brain and cerebrospinal fluid (CSF) of MPS IIIB dogs compared to unaffected dogs. LCMS quantification of HS levels (top row) and HS-NRE (bottom row) in homogenized cerebral grey matter **(A)** and CSF **(B)** from MPS IIIB (circles, grey bars) and unaffected dogs (triangles, empty bars) at different ages from 1 to 12 months old. Levels in cerebral grey matter were normalized to wet brain mass. Bars illustrate the mean (horizontal line) and standard deviation of data for time points at which samples from more than two dogs were analyzed. Data points with a horizontal line through them but no bar represent more than one sample was analyzed but the variability was extremely small. Data points with no horizontal line indicate a sample from a single animal was analyzed. See Supplementary Table 1 for raw data.

The accumulation over time of non-degraded HS polysaccharide and oligosaccharide chains was quantified in MPS IIIB dogs at 1, 2, 3, 6, 9, and 12 months of age. Total HS, as defined by the sum of the internal disaccharides of HS, and the MPS IIIB-specific non-reducing end of HS (the substrate of NAGLU enzymatic activity) were measured in brain tissue (cerebral grey matter), CSF, plasma, and urine (Fig. 1, Supplementary Figure 1, Individual dog IDs presented in Table 1). Unaffected dogs had low levels of HS and HS-NRE in brain tissue, CSF, plasma, and urine at all time points tested (Fig. 1, Supplementary Figure 1). In contrast, levels of total HS and HS-NRE in brain, CSF, plasma and urine of MPS IIIB dogs were markedly increased over levels in unaffected dogs (Fig. 1, Supplementary Figure 1). In the cortex of MPS IIIB dogs, high levels of HS and HS-NRE were detected at 1 month of age (the earliest time point tested), and levels of total HS increased over time and began to plateau by about 12 months of age (Fig. 1A). In the CSF of MPS IIIB dogs, levels of total HS and HS-NRE decreased over time, reaching a steady state of approximately 17-fold over levels in unaffected dogs by approximately 6 months of age (Fig. 1B).

**Table 1:** Individual animals examined for SensiPro analysis.

Animal ID	Phenotype	Age (Months)	CSF HS (pmol/0.6 ul)	CSF HS NRE (pmol/0.6 ul)	CNS HS (pmol/mg)	CNS HS-NRE (pmol/mg)	Urine HS (pmol/nmol creatinine)	Urine HS NRE (pmol/nmol creatinine)	Plasma HS (pmol/0.6 ul)	Plasma HS-NRE (pmol/0.6 ul)
67	Unaffected	1					3.06	0.00	4.71	0.00
123	Unaffected	1	16.87	0.1						
162	Unaffected	2	2.82	0			4.46	0.09	9.10	0.00
163	Unaffected	2	1.16	0			2.12	0.09	10.27	0.00
204	Unaffected	2	2.66	0				0.04	0.01	0.30
58	Unaffected	3			8.58	2.60				
104	Unaffected	3	2.18	0			2.47	0.00	7.37	0.02
167	Unaffected	3	1.84	0	10.14	0.00	4.43	0.05	6.50	0.00
168	Unaffected	3	1.94	0	11.19	0.00	3.48	0.13	7.62	0.00
97	Unaffected	9	2.29	0			1.27	0.01	3.77	0.00
100	Unaffected	9	2.38	0			1.57	0.00	3.70	0.00
93	Unaffected	12					1.05	0.00	4.35	0.00
99	Unaffected	12					5.58	0.04	3.46	0.00
434	Unaffected	12			10.34	0.00	0.76	0.00	2.24	0.00
178	MPS IIIB	1	82.09	3.12	84.23	9.87	305.50	7.14	53.81	0.77
410	MPS IIIB	1	91.65	2.36	63.11	17.23	398.03	6.95	37.50	0.42
427	MPS IIIB	1	79.16	2.13	90.09	1.49			29.34	0.34
376	MPS IIIB	2	40.24	1.13			148.46	3.33	38.89	0.40
397	MPS IIIB	2	36.46	0.93			210.00	4.43	37.69	0.45
425	MPS IIIB	2	74.62	2.26			221.79	4.77	38.93	0.54
155	MPS IIIB	3			58.40	9.86				
174	MPS IIIB	3	44.25	1.55	133.16	14.79	85.85	1.88	40.63	0.41
179	MPS IIIB	3	58.74	2.22	125.50	13.62	70.85	1.62	33.08	0.43
412	MPS IIIB	3	39.11	1.11			118.97	2.53	44.48	0.39
346	MPS IIIB	6	35.81	0.94	182.17	14.28	147.56	3.36	44.67	0.61
340	MPS IIIB	9	30.02	0.73					37.69	0.38
302	MPS IIIB	12	31.45	0.73	188.53	9.75			41.93	0.41
326	MPS IIIB	12	35.29	0.76	240.75	14.05	79.95	1.30	281.37	2.04
328	MPS IIIB	12	35.83	0.86	194.16	8.94	33.70	0.59	173.61	1.30

Individual animal IDs, ages and SensiPro data collected, including levels of heparan sulfate (HS), heparan sulfate non-reducing ends (HS-NRE) in CNS tissue, cerebrospinal fluid (CSF), urine and plasma. Blank cells indicate that no data was collected.

Increased sulfation of HS occurs in mouse models of MPS IIIB and other MPS disorders^18^. To assess whether the composition of the HS changes over time in MPS IIIB dogs, we quantified the extent of the distinct sulfation modifications (2-O, 6-O, and N-sulfation) as well as the abundance of *N*-acetylated glucosamine. We found that the abundance of 2-O sulfated and N-sulfated HS subtly increased while the abundance of *N*-acetylated HS subtly decreased over time in brain tissue of MPS IIIB dogs (Supplementary Figure 2A). The composition of HS in CSF remained consistent over the time period measured (Supplementary Figure 2B). In summary, MPS IIIB dogs have high levels of HS and HS-NRE in several tissues including brain and CSF. These levels are consistently higher in MPS IIIB dogs than unaffected dogs, even at 1 month of age, the earliest time point evaluated. These results demonstrate a way to directly measure the accumulation of NAGLU substrates (HS and HS-NRE) by LCMS in the brain and in fluids which is easily monitored in the clinic (CSF, plasma, and urine) and provides a tool to measure the primary biochemical activity of NAGLU and its correction with therapy.

## MPS IIIB disease forebrain pathology

To understand the effects of HS accumulation on brain pathology, changes in brain architecture were evaluated by histology.

### Increased lysosomal storage in the forebrain of MPS IIIB dogs

**Figure 2:**
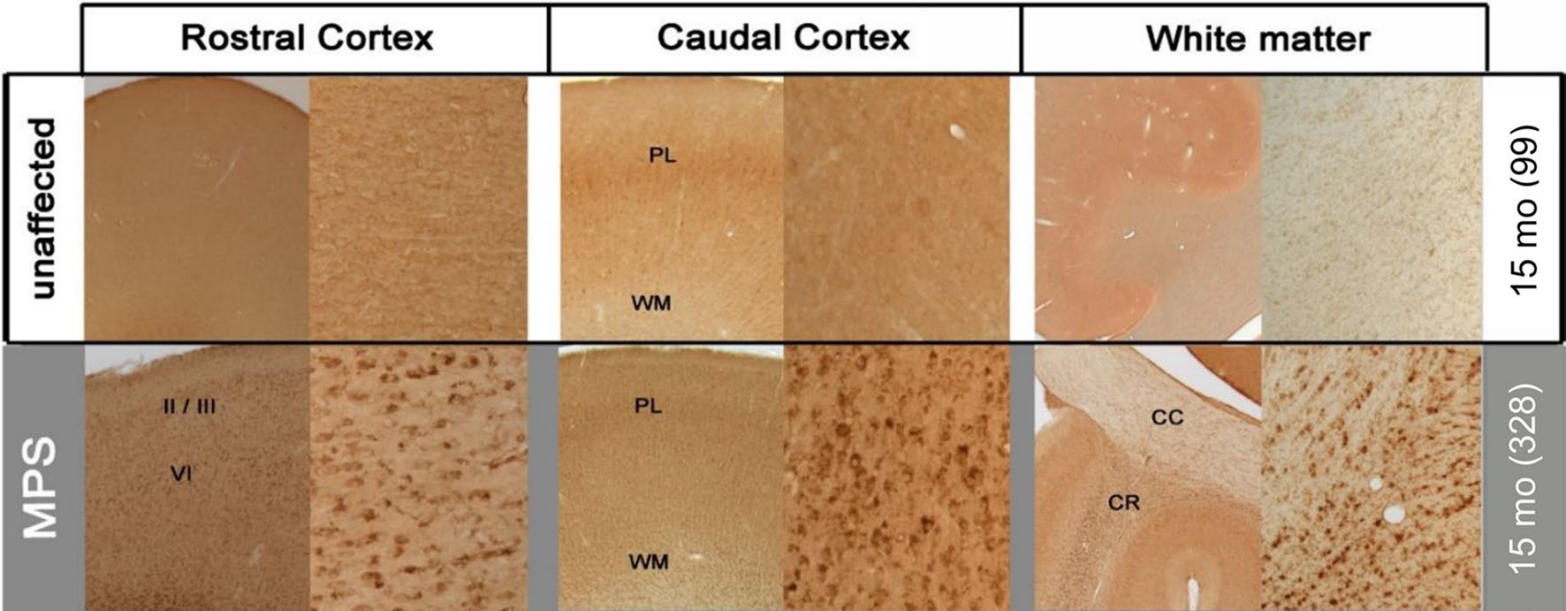
MPS IIIB dogs have increased LAMP1 staining in cortex and white matter. In the area of the cerebral cortex, 15-month-old MPS IIIB dogs (MPS—grey section) display pronounced LAMP1 immunostaining in two distinct layers (II/III and VI) that changes into a more pan-laminar (PL) distribution as sections become more caudal. For each region, the right panel is a macroscopic image whereas the left panel is a 20× magnification. In MPS IIIB, pronounced white matter (WM) LAMP1 immunostaining within corona radiata (CR) was also observed, whereas the corpus callosum (CC) contains much less LAMP1 immunostaining. Numbers next to ages indicate animal ID number.

Lysosomal membrane protein-1 (LAMP1) has been used as a surrogate marker of lysosomal storage burden and has been shown to indicate the size of the lysosomal compartment, despite only being a proxy measure^10^. Tissue from the forebrain of MPS IIIB dogs 15 months of age showed increased LAMP1 staining over the corresponding unaffected controls (Fig. 2). Due to the diffuse background staining within the neuropil of the oldest available MPS IIIB dogs (data not shown), increased LAMP1 staining was most evident in 15-month-old MPS IIIB dogs compared to age-matched unaffected dogs (Fig. 2). A dense accumulation of punctate LAMP1 immunoreactivity was present to different extents in the cytoplasm of individual cells. These cells predominantly had neuronal morphology, although other smaller cells, presumed to be glia, also contained intense LAMP1 immunoreactivity. This staining of individual cells also revealed a series of different regional patterns of LAMP1 immunostaining within the forebrain of 15-month-old MPS IIIB dogs.

In the cortex of 15-month-old MPS IIIB dogs, immunostaining for LAMP1 varied in intensity across the cortical laminae, and at different levels of the brain (Fig. 2). Rostrally, LAMP1 immunostaining was darker within cells in both superficial (II/III) and deeper laminae (VI), but was less intense in the other laminae. In more caudal regions of cortex, this distinction was less apparent with a more even, pan-laminar staining of cells (Fig. 2). Within the white matter, there was also a stark contrast in the distribution of LAMP1 staining; the white matter of the corona radiata that lies directly under cortical grey matter had more intense LAMP1 immunoreactivity than the adjacent white matter of the corpus callosum, where much less LAMP1 staining was evident.

**Figure 3:**
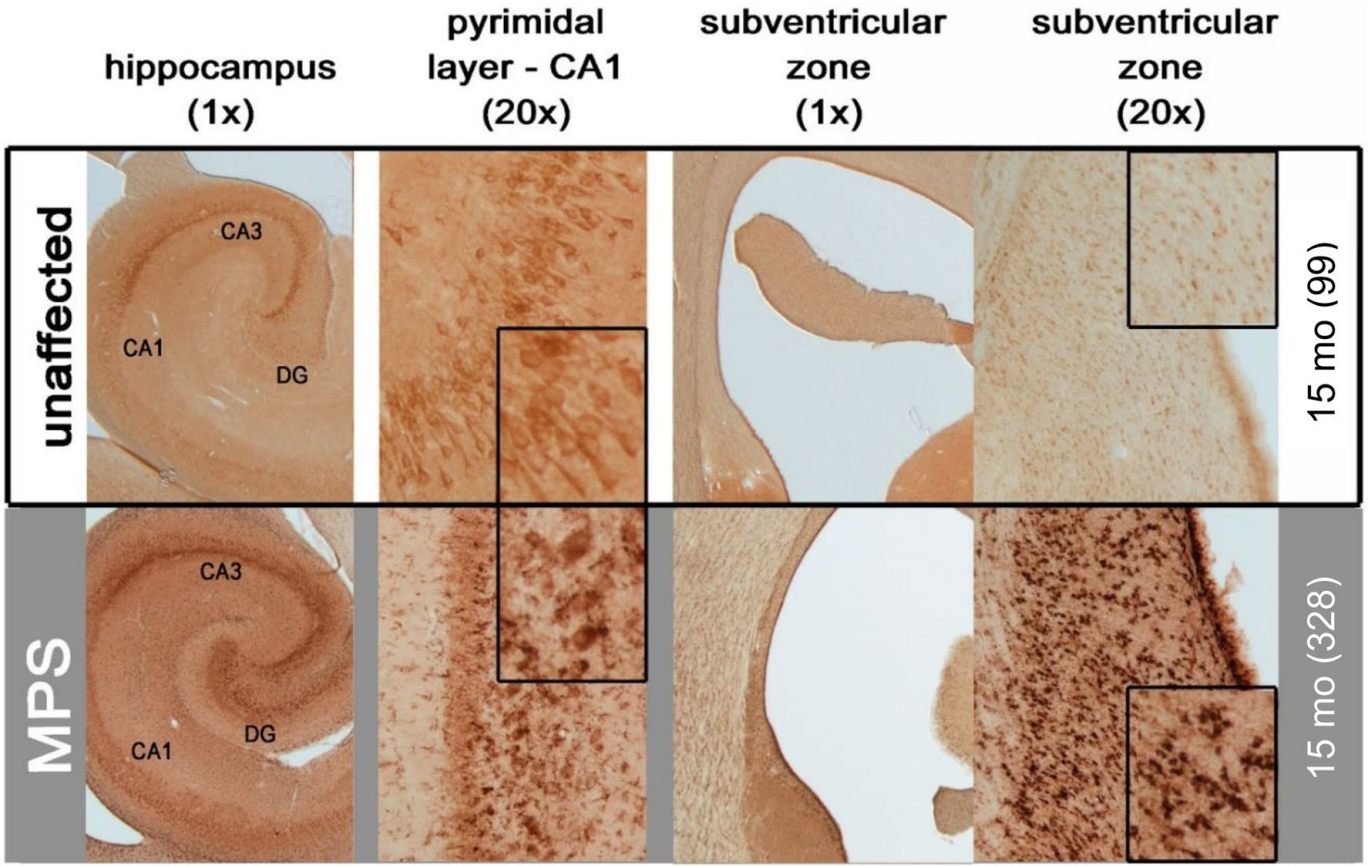
Increased lysosomal storage in hippocampus of MPS IIIB dogs. LAMP1 immunostaining revealed increased lysosomal storage throughout the hippocampus of 15-month-old MPS IIIB dogs (MPS—grey background section), including dentate gyrus (DG), CA3 (CA3), and CA1 (CA1). There was also increased LAMP1 immunostaining in the neurogenic region of the subventricular zone and adjacent white matter. Numbers next to ages indicate animal ID number*.*

**Figure 4:**
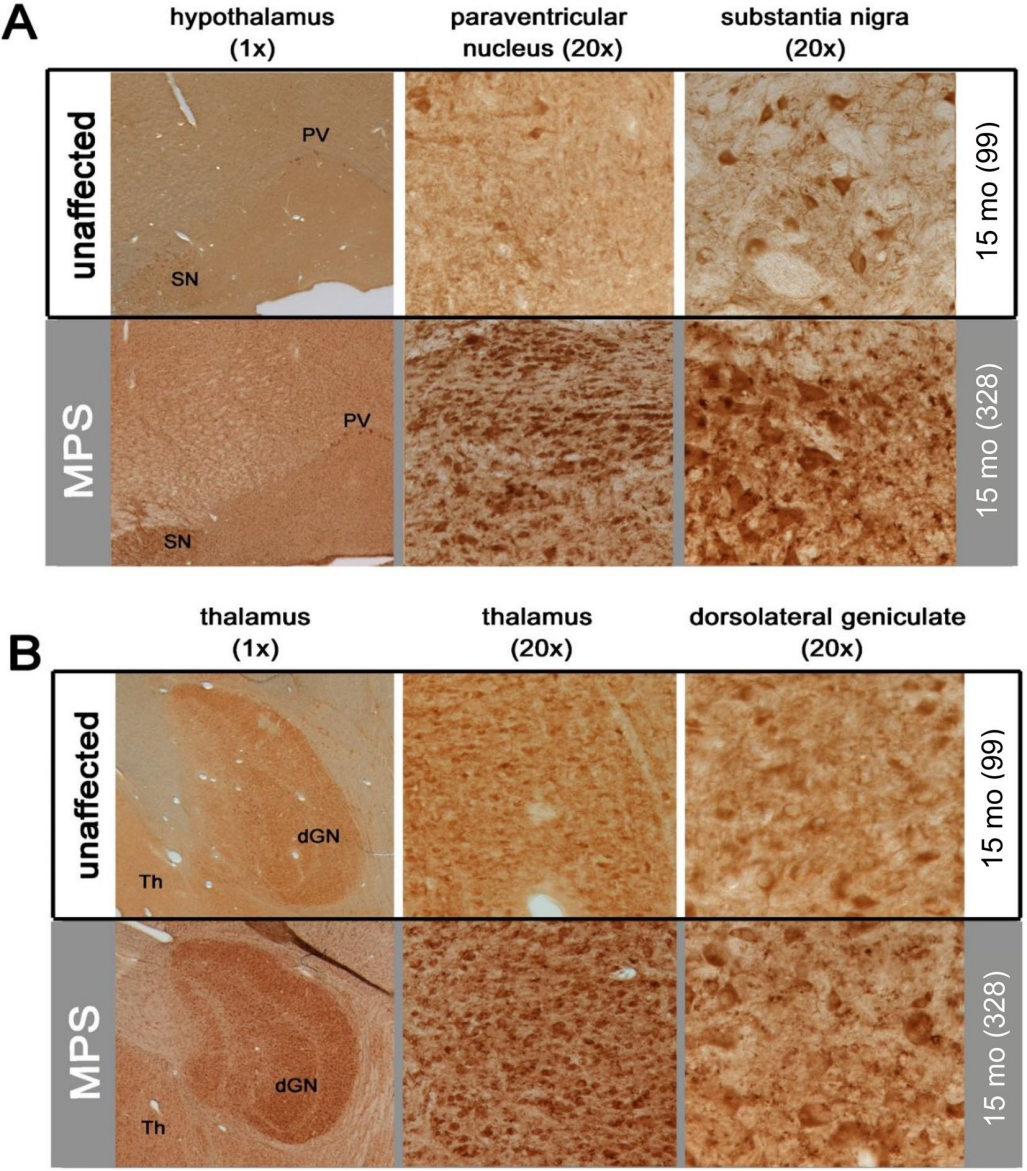
Increased lysosomal storage in hypothalamus and thalamus of MPS IIIB dogs. **(A)** LAMP1 immunostaining reveals pronounced enlargement of the lysosomal compartment in the hypothalamus of MPS IIIB dogs (MPS—grey background), as revealed by more intense LAMP1 immunoreactivity. LAMP1 immunostaining was increased diffusely throughout the hypothalamus in diseased MPS IIIB animals compared to unaffected controls. Large, darkly stained neurons were evident in the paraventricular nucleus (PV—1×), as is evident at higher power (20×). The nearby substantia nigra (SN) of MPS IIIB dogs also contained many large, darkly LAMP1 immunostained neurons compared to control (20×). **(B)** The thalamus (Th) of MPS IIIB dogs generally exhibited darker LAMP1 immunostaining; the dorsolateral geniculate nucleus (dGN) appeared to contain especially intense immunostaining for this marker. This was largely due to intense LAMP1 immunoreactivity within the neuronal cytoplasm (dGN—20×) but also staining within the neuropil, presumably within dendritic processes (dGN—20×). Numbers next to ages indicate animal ID number.

Hippocampal neurons displayed subfield-specific LAMP1 staining in both unaffected and MPS IIIB dogs. This immunoreactivity was much darker in pyramidal neurons of CA3 rather than CA1 of MPS IIIB dogs and also within granule neurons of the dentate gyrus (Fig. 3). There was also staining of LAMP1 with what appeared to be glia scattered through the hippocampal neuropil of 15-month-old MPS IIIB dogs that was not present in unaffected control dogs (Fig. 3). MPS IIIB dogs also had markedly more LAMP1 immunoreactivity throughout the thalamus and hypothalamus, with much more staining within morphologically identified glia, as well as more numerous large, darkly LAMP1-stained neurons in both structures (Fig. 4). In the hypothalamus, the neurons in the paraventricular nucleus seemed to have a particularly increased intensity of LAMP1 staining, as did the nearby substantia nigra. Although unaffected control dogs displayed strong LAMP1 staining in neurons within the substantia nigra pars compacta, these nigral neurons appeared to be enlarged in MPS IIIB dogs. This morphological appearance is indicative of pronounced storage accumulation, consistent with more intense LAMP1 immunostaining in these affected dogs. In addition, other cell types (likely microglia) were also stained in this brain region of MPS IIIB dogs. In the thalamus, the dorsolateral geniculate nucleus had robust LAMP1 staining in both MPS IIIB and unaffected dogs, though staining in neurons of MPS IIIB animals was much darker, as was background staining of the neuropil.

### MPS IIIB dogs do not display cortical atrophy

Previous data from MPS IIIB mice revealed no evidence of significant cortical atrophy^9^. Given that mice may not live long enough to develop the full extent of cortical pathology, we evaluated the presence of cortical atrophy in MPS IIIB dogs by measuring cortical thickness in the most severely affected MPS IIIB dogs ≥ 20 months of age. Measurements were obtained from the somatosensory cortex, a representative region of the sensory cortex that is most severely affected in large animal models of similar neuropathic lysosomal disorders^19^. There was no significant difference in the thickness of the rostral suprasylvian gyrus (Supplementary Figure 3) between this oldest cohort of MPS IIIB dogs and their age-matched unaffected counterparts indicating that even in advanced stages of the disease, cortical atrophy does not occur in this canine MPS IIIB model.

### MPS IIIB dogs display qualitative increases in microglial activation in the cerebral cortex

**Figure 5:**
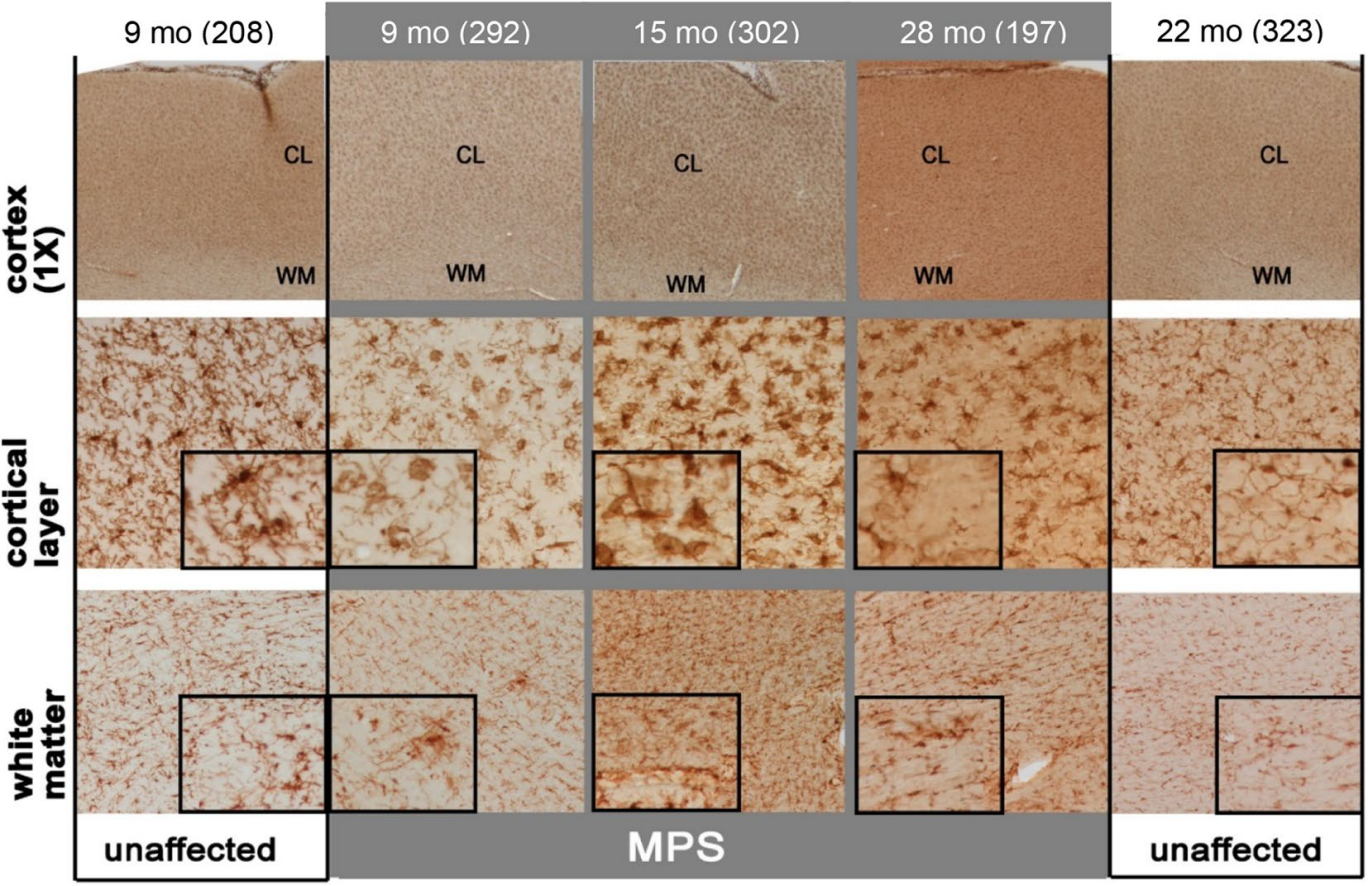
Progressive microglial activation in the cortex of MPS IIIB dogs. Qualitative assessment of immunostaining for the microglial marker Iba1 revealed differences in the distribution and intensity of immunoreactivity in both the cortical layers (CL) and white matter (WM) of the cerebral cortex spanning from 9 to ~ 28 months in the distribution and staining in the cerebral cortex of MPS IIIB dogs (MPS—grey background). MPS IIIB dogs, in comparison to age-matched unaffected controls (unaffected—white backgrounds), at early (9 month) and late stages of the disease (28 months) show differences in microglial morphology and number (magnified inset), and display a larger cell body with thicker processes typical of activated microglia. An increase in microglial activity in the white matter of diseased MPS IIIB animals was mainly present in the two oldest cohorts. This difference was especially evident in the white matter adjacent to the lateral ventricles, including the presence of clusters of activated microglia (see inset). Numbers next to ages indicate animal ID number.

Despite their lack of overt cortical neurodegeneration, MPS IIIB mice display pronounced and progressive microglial activation^9,20^. Typical markers of microglial activation, such as CD68, often do not have reactivity in canine tissue. Instead, immunostaining was performed with Iba1, a microglial marker that is present in quiescent cells and is increased with microglial activation^21^. Marked differences in microglial activation and morphology were observed between unaffected and MPS IIIB dogs. Such changes were evident by 3 months of age and became more pronounced over time (Fig. 5).

**Figure 6:**
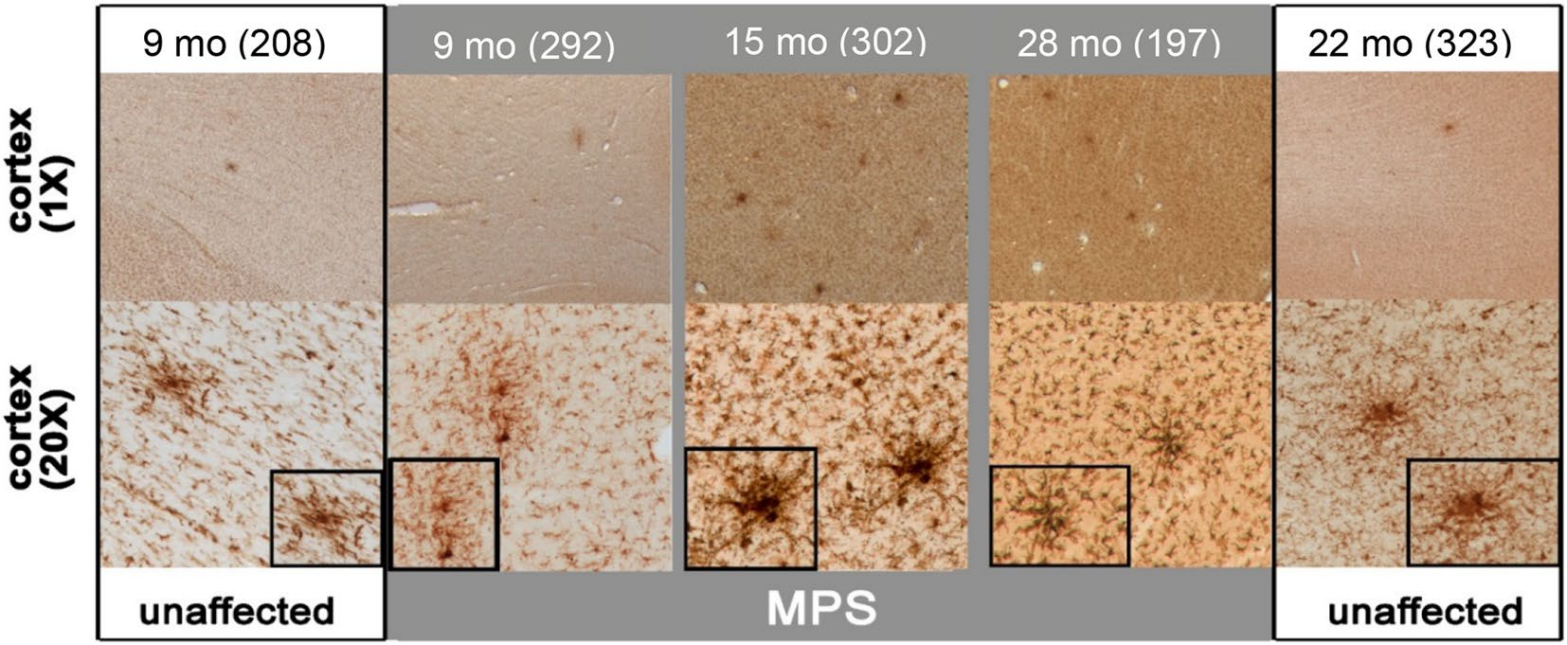
MPS IIIB forebrains have more frequent clusters of activated microglia. At all ages, MPS IIIB dogs (MPS—grey background) had a greatly increased frequency of clusters of intensely Iba1 immunostained activated microglia. These clusters were especially frequent in the diseased 9- and 15-month-old forebrains. Also apparent were changes in microglial morphology in MPS IIIB brains, with larger darkly stained microglia present compared to unaffected brains (see insets). Numbers next to ages indicate animal ID number*.*

Surveying the distribution of Iba1 immunoreactivity across brain regions of MPS IIIB dogs revealed no particular focal area of microglial activation. For example, within the cortical mantle, Iba1 immunoreactivity was uniformly increased across the depth of all cortical regions of MPS IIIB dogs, with no greater increase in any individual lamina (Fig. 5, upper panels). Similar but less pronounced changes were evident within the underlying white matter (Fig. 5, upper panels). A notable exception was the presence of localized clusters of intensely stained Iba1-positive microglia in white and grey matter of MPS IIIB dogs of all ages (Fig. 6). Similar microglial clusters were also observed in unaffected dogs, but to a lesser extent. The distribution of these clusters did not appear to favor any specific brain region, but occurred with a similar frequency at all rostro-caudal levels of the brain in both cortical and subcortical structures. Equally, there was no evidence that the clusters associated with blood vessels and the frequency of the clusters did not appear to correlate with disease progression.

There were also clear differences in microglial morphology between unaffected and MPS IIIB dogs, which became more pronounced in MPS IIIB dogs with age. In unaffected animals, the many thin ramified Iba1-positive processes characteristic of quiescent or resting microglia gave the background of the neuropil a “lacy” appearance (Fig. 5, cortical layer inset), an appearance that was less apparent in MPS IIIB dogs. Morphological changes in microglia in MPS IIIB dogs reflected their activated state, being not only more intensely stained than cells in unaffected controls, but also with fewer thickened processes and an enlarged cell soma, typical of activated microglia.

Iba1-positive morphologically active microglia were particularly evident in the cortical mantle of MPS IIIB dogs from 3 months of age and were distributed uniformly throughout the cortex (Fig. 5, and with age morphological differences between Iba1-positive microglia became more pronounced. Twenty-month-old MPS IIIB dogs had increased incidence of distended microglial cell bodies in the cortical neuropil (Fig. 5). Measurements of the cross-sectional area of Iba1-positive microglial soma confirmed that these microglia were significantly larger in MPS IIIB dogs than their wild-type counterparts at 20 months of age or older (Supplemental Figure 2). There was also a characteristic increase in microglial activation in the white matter in MPS IIIB dogs that was not seen in unaffected controls (Fig. 5). Although the morphological changes in white matter microglia were not as pronounced in the cortical grey matter, the soma of these microglia also appeared larger at later stages of disease progression (Fig. 5, inset).

### Cerebellar pathology

The initial characterization of the schipperke dog model of MPS IIIB reported neurologic signs consistent with cerebellar disease, evidence for cerebellar atrophy, and Purkinje neuron loss^17^. To broaden these observations, we assessed the degree of storage burden, glial activation, and neuronal survival in the cerebellum of MPS IIIB dogs.

### Increased lysosomal storage in the cerebellum of MPS IIIB dogs

**Figure 7:**
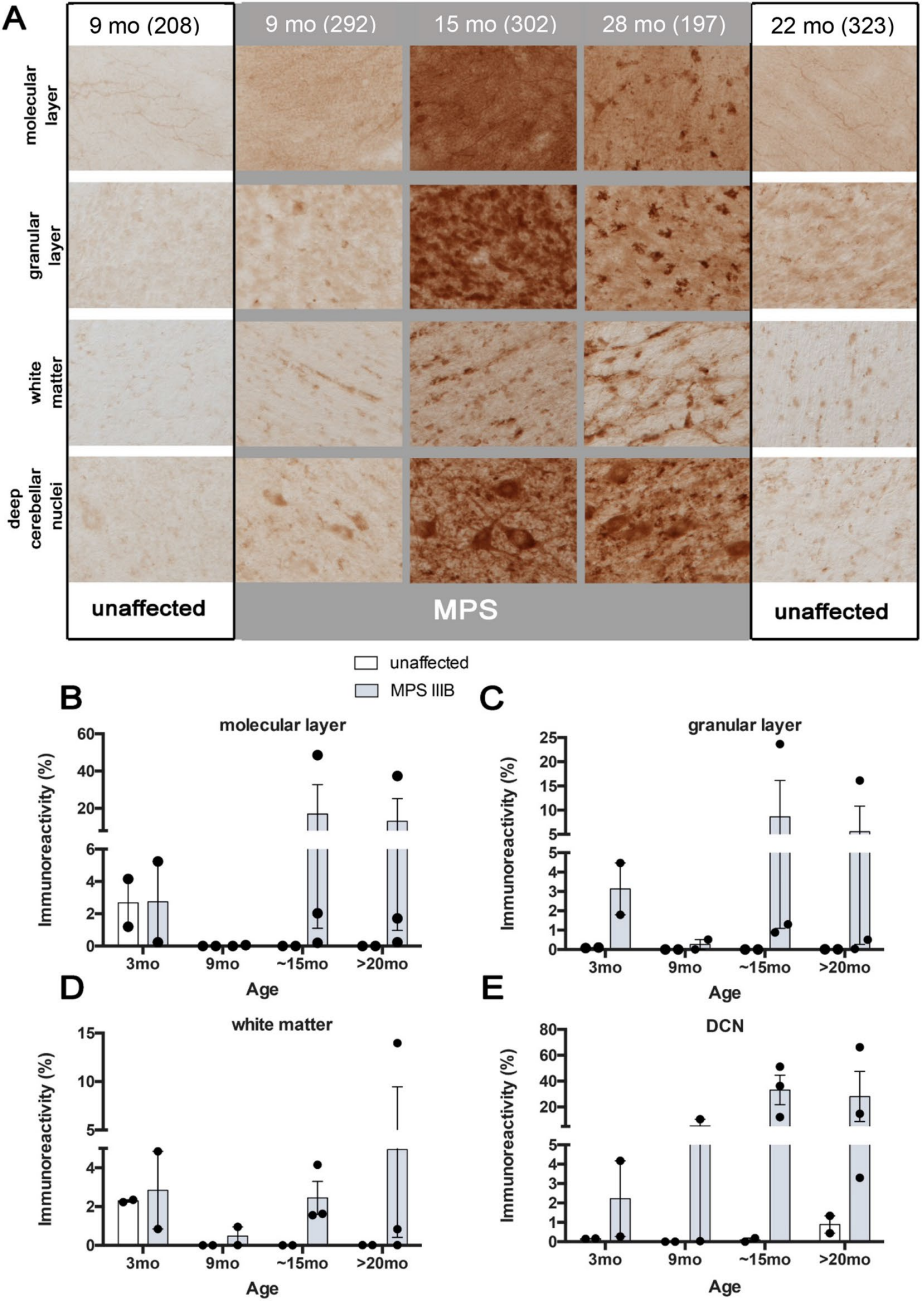
Enlargement of the lysosomal compartment in the cerebellum of MPS IIIB dogs. **(A)** Immunostaining for the lysosomal marker LAMP1 provides a surrogate marker for storage material accumulation and reveals a progressive increase in the size of the lysosome in the cerebellum of MPS IIIB dogs (MPS IIIB—grey) compared to unaffected controls (unaffected—white). These changes, examined using thresholding analysis, were apparent in the molecular layer, the Purkinje cell layer, the cerebellar white matter and the deep cerebellar nuclei (DCN). **(B–E)** Thresholding image analysis of LAMP1 immunostaining confirms the progressive increase in staining intensity for this marker in the cerebellum of MPS IIIB dogs compared to unaffected controls. Markedly increased LAMP1 staining was evident in all regions except the white matter **(D)** by 15 months of age, and in all regions by later stages (~ 28 months). Error bars indicate SEM. Number of animals analyzed (3 mo: unaffected n = 2, MPS IIIB n = 2; 9 mo: unaffected n = 2, MPS IIIB n = 2; 15 mo: unaffected n = 2, MPS IIIB n = 3; > 20 mo: unaffected n = 2, MPS IIIB n = 3). Numbers next to ages indicate animal ID number.

Lysosomal storage burden was greatly increased in several areas of the cerebellum in the schipperke MPS IIIB dogs^17^. In our study, immunostaining of LAMP1, a surrogate marker for storage burden, was increased in MPS IIIB dogs at all ages examined, as compared to age-matched unaffected dogs, although there was significant inter-sample variability in the immunostaining intensity (Fig. 7). In unaffected dogs, LAMP1 staining was very faint, and only appreciable within the characteristically large soma of Purkinje neurons, whose morphology was readily apparent. In contrast, there was dramatically more LAMP1 staining in MPS IIIB dogs. While this increase was evident at all ages examined, the difference between the diseased state and unaffected controls was more obvious by 9 months of age. In these MPS IIIB dogs, LAMP1 not only intensely stained Purkinje neurons and their projections within the molecular layer, but also stained the granule cell layer and DCN, becoming especially pronounced at 15 months of age (Fig. 7). In the oldest MPS IIIB dogs, the pattern of LAMP1 staining changed again, with far fewer stained Purkinje neurons, markedly less staining in the molecular layer, and fewer, more intensely stained cells in the granule cell layer. This change in distribution could reflect the loss of Purkinje neurons and granule cells in MPS IIIB dogs, with more LAMP1 staining in microglia (Fig. 7). These findings suggest that storage burden, as measured by LAMP1 immunostaining, reaches a peak in brains of MPS IIIB dogs before tapering off in later stages of the disease, likely due to cell death of various cell types expressing LAMP1.

### Progressive gliosis in the MPS IIIB cerebellum

The activation of both astrocytes and microglia occurs as a response to cell damage in LSDs such as MPS IIIB^9,10,20^. Using GFAP as a marker, we assessed the extent of astrogliosis in canine MPS IIIB disease.

### Progressive astrogliosis in the cerebellum of MPS IIIB dogs

**Figure 8:**
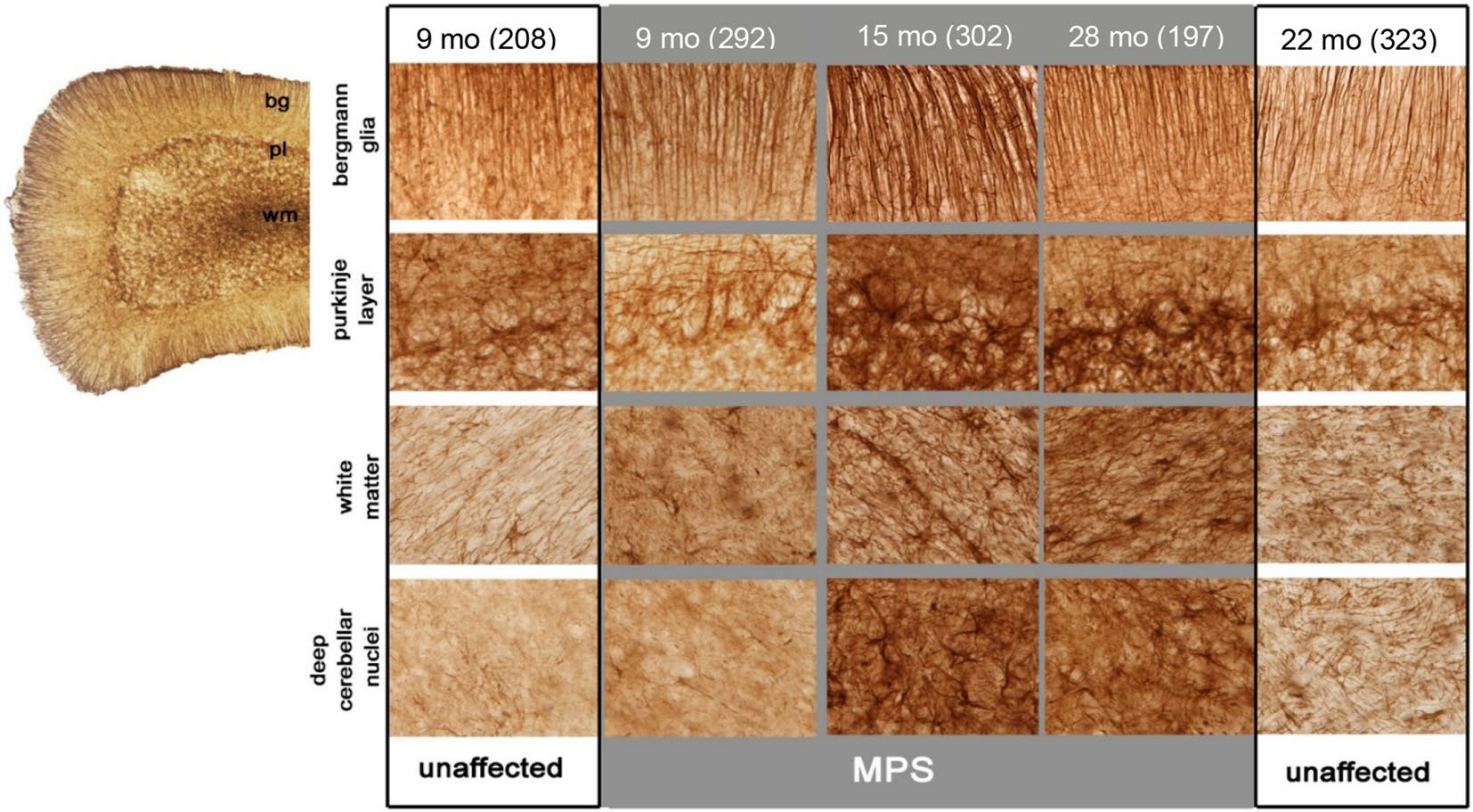
Astrocytosis in the MPS IIIB dog cerebellum. Qualitative assessment of immunostaining for the astrocyte marker GFAP reveal progressive changes from 9 to ~ 28 months in the distribution and staining intensity in the different layers of the cerebellum of MPS IIIB dogs (MPS—grey background). Comparison of MPS IIIB animals to unaffected controls (unaffected—white background) at early (9 month) and late stages of the disease (28 months) shows qualitative differences particularly in the white matter and deep cerebellar nuclei, primarily at later stages. Abbreviations: bg, Bergmann glia layer; pl, Purkinje layer; wm, white matter. Numbers next to ages indicate animal ID number*.*

Across all age groups, GFAP immunostaining was consistently elevated in MPS IIIB dogs compared to the unaffected controls. However, the high inter-animal variability precluded quantitative analyses. However, even unaffected animals at all ages displayed a substantial number of GFAP-positive Bergman glia within most folia, although these cells displayed relatively thin processes (Fig. 8, Bergman glia). This was accompanied in unaffected dogs by relatively little staining of fibrous astrocytes within the cerebellar white matter (Fig. 8, white matter). In contrast, the pattern and intensity of GFAP immunostaining was markedly different in MPS IIIB dogs at all ages examined (Fig. 8, grey), and became more complex with disease progression. Bergman glia in MPS IIIB dogs displayed much thicker processes than in corresponding age-matched unaffected controls (Fig. 8), and a distinctive band of GFAP immunostaining was evident at the interface between granule and molecular layers, marking where Purkinje cells normally sit (Fig. 8, Purkinje layer). This reached its peak intensity in MPS IIIB dogs by 15 months of age, before declining in older MPS IIIB dogs (Fig. 8). This was accompanied by progressively more GFAP immunostaining with increased age within both the granule cell layer, and perhaps most notably within the white matter and DCN of MPS IIIB dogs (Fig. 8). Increased GFAP immunostaining within the white matter was already visible in MPS IIIB dogs from 9 months of age, revealing a distinctive outlining of astrocyte processes around blood vessels within the cerebellar white matter (Fig. 8). These results suggest progressive changes in astrocyte activation in MPS IIIB dogs, specifically around the Purkinje cell layer and cerebellar white matter.

### Microgliosis is most prominent in the DCN in cerebellum of MPS IIIB dogs

**Figure 9:**
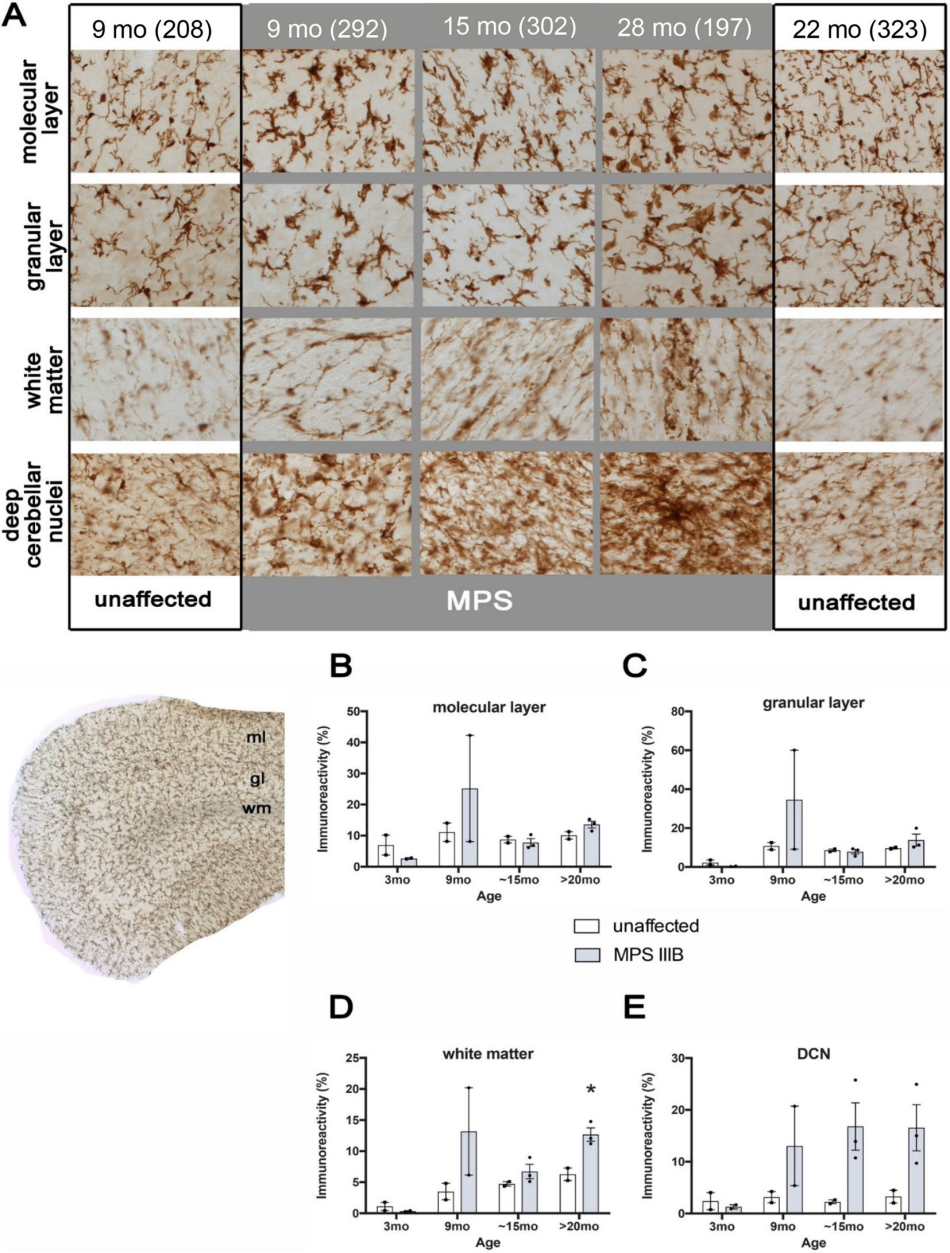
Microglial activation in the MPS IIIB dog cerebellum. **(A)** Immunostaining for the microglial marker Iba1 revealed progressive changes from 9 to ~ 28 months in the distribution and staining in the cerebellum of MPS IIIB dogs (MPS—grey background). MPS IIIB animals, in comparison to unaffected controls (unaffected—white background) at early (9 month) and late stages of the disease (28 months) shows differences in microglial morphology and number, displaying a larger cell body with thicker processes, typical of activated microglia. **(B-E)** Quantification using thresholding image analysis of Iba1 immunostaining confirms the progressive increase in immunostaining intensity in the cerebellum of MPS IIIB dogs compared to unaffected controls (unaffected). Pronounced trends towards increases in Iba1 immunostaining appear in all regions but are significant in the cerebellar white matter **(D)** and deep cerebellar nuclei **(E)** (*p < 0.05). Abbreviations: ml, molecular layer; gl, granular layer; wm, white matter. Error bars indicate SEM. Number of animals analyzed (3 mo: unaffected n = 2, MPS IIIB n = 2; 9 mo: unaffected n = 2, MPS IIIB n = 2; 15 mo: unaffected n = 2, MPS IIIB n = 3; > 20 mo: unaffected n = 2, MPS IIIB n = 3). Numbers next to ages indicate animal ID number.

Iba1 immunoreactivity was much less variable than GFAP staining and revealed changes in morphology and increased Iba1 immunostaining in MPS IIIB dogs compared to the unaffected controls from 9 months of age, and were more pronounced at later stages (Fig. 9). Compared to the lacy appearance of the neuropil in unaffected dogs, Iba1-positive microglia in MPS IIIB dogs displayed fewer, thicker processes and had enlarged cell soma. A distinctive appearance of very large microglia and/or clusters of intensely stained Iba1-positive cells were seen sporadically within both granule and molecular layers of MPS IIIB dogs from 9 months of age. Compared to astrocytosis, microglial activation was less pronounced within the white matter of MPS IIIB dogs, but was still markedly higher than in unaffected dogs, with localised activation evident in the DCN (Fig. 9). Quantification of Iba1 immunoreactivity via thresholding image analysis revealed some inter-animal variability. In particular, one of the 9-month-old MPS IIIB brains had markedly increased Iba1 immunostaining than any other brain of the same age. However, there was a significant increase in Iba1 immunoreactivity in white matter of MPS IIIB dogs at > 20 months of age (Fig. 9D), with a trend starting at 15 months of age. A similar trend in relative Iba1 immunoreactivity was seen in the DCN of MPS IIIB dogs, but not in their cerebellar cortices in either the molecular or granular layers. These results indicate, similarly to the atrophy, much of the cerebellar microgliosis occurs in the white matter. While there were obvious changes in microglial morphology in the cerebellar cortical layers of MPS IIIB dogs, significant changes in Iba1 immunoreactivity were detected only in the white matter and the DCN.

### Loss of neurons and white matter atrophy in the cerebellum of MPS IIIB dogs

**Figure 10:**
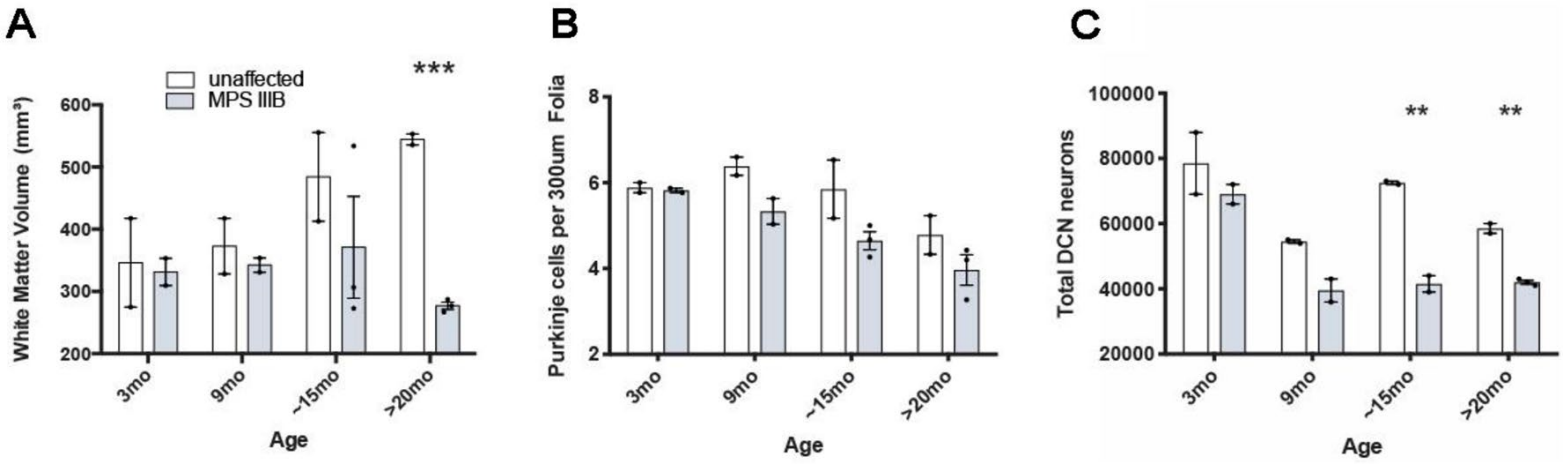
Progressive neuron loss in the cerebellum of MPS IIIB dogs. Statistical significance between unaffected and MPS IIIB dogs is indicated by ** (0.01) and *** (0.001). **(A)** MPS IIIB dogs did not display the progressive increase in white matter volume seen in unaffected dogs, resulting in reduced white matter volume in late stage MPS IIIB dogs. **(B)** Purkinje cell counts revealed progressive loss in MPS IIIB dogs. **(C)** Unbiased stereological counts revealed progressive loss of neurons in the deep cerebellar nuclei (DCN) of MPS IIIB dogs; neuron numbers at 15 and > 20 months of age were significantly decreased compared to those found in unaffected dogs. Examining just the data from MPS IIIB dogs (grey bars) revealed a significant decrease in DCN neurons at 9, 15, and 28 months compared to 3 months of age. Error bars indicate SEM. Number of animals analyzed (3 mo: unaffected n = 2, MPS IIIB n = 2; 9 mo: unaffected n = 2, MPS IIIB n = 2; 15 mo: unaffected n = 2, MPS IIIB n = 3; > 20 mo: unaffected n = 2, MPS IIIB n = 3). Numbers next to ages indicate animal ID number.

Purkinje cells and neurons in the DCN provide the major efferent output from the cerebellum, indicating their importance for cerebellar functions. Loss of these cells was analyzed by Nissl staining and the sections were used to evaluate white matter volume loss and cerebellar cortical thickness (Fig. 10). The thickness of cerebellar molecular and granular layers was measured in the posterior lobe of the cerebellum both separately and together, but revealed no significant change in the thickness of any cerebellar cortical layer in MPS IIIB dogs at any age (data not shown). Cerebellar white matter volume changes were apparent in MPS IIIB dogs from 15 months of age, but became statistically significant at > 20 months of age when the volume was decreased by more than 50% (Fig. 10A). There was an increase in white matter volume in unaffected dogs over time, potentially due to ongoing myelination during postnatal maturation, which was not evident in MPS IIIB dogs, resulted in a significantly smaller volume in the oldest MPS IIIB dogs (Fig. 10A). A marked decrease in Purkinje cells (15–20%) was observed in the posterior lobe of the cerebellum of MPS IIIB dogs at all ages when compared to age-matched unaffected controls. Though this did not reach statistical significance, the trend in Purkinje cell loss with disease progression was evident (Fig. 10B). Optical fractionator counts of Nissl-stained DCN neurons showed a pronounced loss of these cells in MPS IIIB dogs by 9 months of age, which then remained stable at later stages of disease (Fig. 10C). Compared to unaffected controls, the loss of DCN neurons in MPS IIIB dogs became statistically significant after 15 months of age. However, compared to 3- month-old MPS IIIB dogs, the loss of DCN neurons was statistically significant from 9 months onwards (Fig. 10C). Taken together, these data indicate that cerebellar atrophy occurs primarily in the white matter, and that there is progressive loss of DCN neurons that has not been reported previously.

## Discussion

In this study, we assessed the development of disease pathology in the canine model of MPS IIIB before they display any signs, by evaluating biochemical and histopathological measures of lysosomal storage accumulation, neurodegeneration, and atrophy.

The changes in levels of both HS and MPS IIIB-specific HS-NRE were evaluated in cortical grey matter, CSF, plasma, and urine from MPS IIIB dogs. Even by 1 month of age, HS and HS-NRE levels in tissues and body fluids from MPS IIIB dogs were higher than the levels in the corresponding samples from unaffected dogs. However, the kinetics of HS and HS-NRE accumulation differ across the four tissues or body fluids. While levels of HS and HS-NRE in cortical grey matter of MPS IIIB dogs increase over time, levels of HS and HS-NRE in CSF and urine of MPS IIIB dogs were higher at 1 month of age than at subsequent time points. Despite this early decrease, levels of HS and HS-NRE in CSF and urine of MPS IIIB dogs at these later time points remain elevated in comparison to the levels present in unaffected dogs of any time point. High levels of urinary GAGs have been reported previously in healthy newborn humans and have been suggested to be associated with increased turnover of extracellular matrix during development^22–24^. Elevated levels of HS and HS-NRE in the CSF and urine of 1-month old MPS IIIB dogs could be indicative of the ongoing brain growth and increased turnover of extracellular matrix that occurs during this time period^25^. Further studies that include additional WT and MPS IIIB dogs at younger ages will be needed to fully understand the mechanism driving the early elevated levels of HS and HS-NRE in MPS IIIB dogs. Any future studies of potential therapies that initiate prior to HS and HS-NRE levels reaching steady state in the CSF of MPS IIIB dogs will need to distinguish between the developmentally associated decrease in HS levels, and that due to any therapeutic benefit at these early time points.

Disease-related pathology illustrated by increased intensity of LAMP1 immunostaining was observed in multiple brain regions of preclinical MPS IIIB dogs and was accompanied by a progressive increase in microgliosis (cortex and cerebellum) and astrogliosis (cerebellum). The distribution of these changes is similar to that of histological changes seen in the brain of an MPS IIB patient^26^, suggesting that the dog model recapitulates this aspect of the human disease.

Pathological evaluation of the cerebellum confirmed it to be the most severely affected brain region in preclinical MPS IIIB dogs, displaying neuropathological changes that are evident relatively early in disease progression. One of the most prominent and previously unreported findings within the cerebellum was a substantial 50% loss of DCN neurons. This loss occurred relatively early in disease progression (present by 9 months of age) and did not appear to progress further, indicating that the majority of DCN neurodegeneration occurs in early stages of the disease. As such, it may be important to look in patients for symptoms that could potentially be related to this DCN loss. There was also significant cerebellar white matter atrophy in preclinical MPS IIIB dogs, which had almost half the volume compared to unaffected dogs. This may be due to axon loss or a demyelination process resulting from a degeneration or dysfunction of oligodendrocytes or defective myelination during post-natal development. However, we observed an increase in white matter volume in unaffected dogs suggestive of ongoing myelination during this time in development. The degree of Purkinje cell loss in these MPS IIIB dog samples was relatively small and not significantly different from controls, as was previously reported in older animals^17^.Quantitative analysis revealed clear signs of increased storage burden in cerebellar cortical layers of MPS IIIB dogs in addition to DCN and cerebellar white matter. Taken together these observations indicate that the DCN are the primary pathological target within the MPS IIIB cerebellum, exhibiting pronounced neurodegeneration and atrophy of surrounding white matter tissue.

The time-dependent accumulation of HS in the forebrain and progressive increase in LAMP1 staining in both the forebrain and cerebellum in MPS IIIB dogs indicate that MPS IIIB disease pathology clearly occurs prior to the onset of clinical signs in animals. Such signs only occur subsequent to 20 months of age, with all but the most severely affected dogs examined in this study being younger than this. While disease-related pathology is present throughout the brain, the cerebellum is more dramatically affected than other regions in preclinical MPS IIIB dogs. Although the loss of Purkinje neurons has been documented previously in the schipperke MPS IIIB dogs^17^, our current findings provide the first evidence for pronounced pathology within deeper structures of the cerebellum that include the white matter and DCN.

While human MPS IIIB disease is thought to primarily affect cortical brain regions, the extent to which the cerebellum is affected and plays a role in the disease symptoms has not been thoroughly investigated. Interestingly, early stage MPS IIIB disease shares many symptoms with autism despite the different etiologies and MPS IIIB is often misdiagnosed as autism^27^. Similar symptoms include motor dysfunction, aggressiveness, hyperactivity, and language and communication deficits^27^. In addition to changes in brainstem volume and cortical regions, autism is associated with cerebellar atrophy and dysfunction, which may cause motor dysfunction as well as some non-motor symptoms such as deficits in language, communication, social interactions, stereotyped behavior, and cognitive dysfunction^28,29^. Similar symptoms are observed in MPS IIIB patients, suggesting that the cerebellar pathology seen in the MPS IIIB dogs may have relevance for early stages of human MPS IIIB disease. Later stages of the human MPS IIIB disease, which ultimately result in a vegetative state, are likely to involve more widespread neurodegeneration.

Our study reveals the dynamics of the onset and progression of neuropathology in this large animal model of MPS IIIB disease, before the onset of disease signs. We describe a series of markers that can be used for assessing therapeutic benefit in future studies, focusing on well-defined neuropathological and biochemical endpoints including the direct measure of NAGLU substrate accumulation (HS and HS-NRE), Iba1 as a marker of microglial activation, and LAMP1 as a marker of lysosomal enlargement.

## Materials and methods

### Dogs and tissue extraction

The outbred MPS IIIB canine colony was founded based on a naturally-occurring mutation in schipperke dogs^17^. Animals were genotyped by 7 days old with polymerase chain reaction at the canine *NAGLU* locus, and enzyme deficiency was confirmed by testing their serum enzyme levels^30^. The MPS IIIB dogs and their unaffected counterparts were produced and maintained at Iowa State University’s Department of Animal Science according to guidelines of the USDA and NIH, and under approved protocols of the ISU IACUC. Animals were kept with their dams until weaning, and at 4 months segregated by sex. After weaning, dogs were fed ad libitum. CSF was aseptically collected under full anaesthesia by puncture of the cisterna magna after which dogs were euthanized by intravenous administration of sodium pentobarbital at approximately 1, 2, 3, 6, 9, 12, 15, and 20 months of age, after which encephalectomy was performed by Iowa State University Department of Animal Science staff. The numbers of individual dogs analysed at each age is summarized in Table 1 (SensiPro) and Table 2 (histology). Brains were cut sagittally into hemispheres at the level of the longitudinal fissure, with one half immersion fixed in 10% neutral buffered formalin and the other half frozen and stored at − 80 °C. Tissue samples for HS analysis were derived from frozen hemispheres, which were placed at − 20 °C overnight, with tissue samples dissected directly from these − 20 °C frozen brains. Cortical grey matter was taken from the medial aspect of the precruciate gyrus. Urine and blood samples were collected pre-mortem (serum) or immediately post-mortem (urine).

**Table 2:** Individual animals examined for histological analysis.

ID	Phenotype	Age (months)	IBA1				LAMP1			
			WM	ML	GL	DCN	WM	ML	GL	DCN
670	MPS IIIB	3	0.4	3.8	3.6	4.0	2.2338	1.1984	0.0810	0.1509
676	MPS IIIB	3	1.8	10.2	1.0	0.7	2.3489	4.1601	0.1048	0.1681
340	MPS IIIB	9	20.2	42.3	60.1	20.7	0.2562	1.5429	0.1434	1.9971
292	MPS IIIB	9	6.2	8.1	9.2	5.4	0.0022	0.0094	0.0000	0.0002
302	MPS IIIB	15	5.1	6.8	8.7	10.7	1.2043	0.0007	10.7174	24.9239
326	MPS IIIB	16.6	9.0	10.3	9.4	25.8	0.3975	0.0003	0.4723	12.3951
328	MPS IIIB	16.6	6.0	6.2	5.6	13.9	0.5270	0.0001	0.3418	2.0102
227	MPS IIIB	25	11.1	14.0	10.2	9.7	6.4790	1.5660	7.9240	34.7327
200	MPS IIIB	28	12.0	11.5	11.3	15.0	0.1783	0.0004	0.1189	2.5216
197	MPS IIIB	28	14.8	15.3	19.9	24.9	0.0003	0.0005	0.0094	0.2200
674	Unaffected	3	0.2	2.9	0.2	1.7	4.8459	0.2378	4.4699	0.2684
675	Unaffected	3	0.5	2.4	0.4	0.9	0.8453	5.2396	1.7916	4.1729
208	Unaffected	9	2.2	8.2	8.9	2.1	0.0006	0.0011	0.0002	0.0002
210	Unaffected	9	4.8	14.0	12.6	4.2	0.0000	0.0078	0.0023	0.0000
93	Unaffected	15	4.4	9.8	9.2	2.6	0.0000	0.0009	0.0001	0.0006
99	Unaffected	15	5.1	7.7	7.9	1.9	0.0003	0.0000	0.0012	0.0021
132	Unaffected	22	7.2	8.9	9.2	4.5	0.0004	0.0000	0.0092	0.1325
323	Unaffected	22	5.3	11.3	10.2	2.0	0.0019	0.0000	0.0043	0.0400

Individual animal IDs, ages and thresholding image analysis data collected from the cerebellum for the microglial marker IBA1 and for lysosomal membrane associated protein 1 (LAMP1). Regions measured include cerebellar white matter (WM), molecular layer (ML), granule cell layer (GL) and deep cerebellar nucleus (DCN).

### Quantification of total and disease-specific heparan sulfate

Total HS and MPS IIIB-specific HS-NRE in homogenized brain tissue (cerebral grey matter), CSF, serum and urine from approximately 1, 2, 3, 6, 9, and 12-month-old unaffected or MPS IIIB dogs (exact ages of dogs for whom brain tissue, CSF, plasma, and urine were analyzed varied by sample availability) were quantified using the HS-NRE liquid chromatography-mass spectrometry (LCMS) assay (SensiPro) as described previously^2,10,31^. Briefly, HS from the samples was purified by anion exchange chromatography and digested with heparan lyases (IBEX Technologies). The HS fragments were tagged with isotropically labelled aniline by reductive amination and quantified by LCMS. Total HS (internal disaccharides) and the MPS IIIB-specific HS-NRE (*N*-acetylglucosamine-2-sulfated iduronic acid-N-sulfated glucosamine [GlcNAc-IdoA2S-GlcNS], referred to as A0I2S0) are expressed as pmol/mg brain wet weight, pmol/0.6 μl CSF or plasma, or pmol/nmol creatinine in urine.

### Histological preparation of brain tissue

Brains were stored in formalin for extended periods before histology was performed. Before beginning histology, brain hemispheres were submerged in cryoprotectant, 30% sucrose in Tris-buffered saline (TBS: 50 mM Tris, 150 mM NaCl, pH 7.6) for at least two weeks. The forebrain was then separated from the cerebellum and cut perpendicular to the long axis of the brain into three rostrocaudal blocks that were sectioned individually. Blocks were coated with Lipshaw M-1 cryomatrix resin (ThermoFisher Scientific, Leicestershire, UK) and the forebrain cut coronally on a freezing microtome at 50 µm (Microm HM430, ThermoFisher Scientific), with the cerebellum cut sagittally. Sections were collected into 24 well plates containing a lab-derived cryoprotectant solution (TBS containing 30% ethylene glycol, 15% sucrose, and 0.05% sodium azide), and stored at 4 °C until staining was performed.

### Nissl staining

In order to visualize tissue cytoarchitecture, regularly spaced sections were Nissl stained with cresyl violet, as described previously^32^. Briefly, every forty-eighth 50 µm section through each block was mounted onto microscope slides previously double coated with gelatin-chrome allum (ThermoFisher Scientific). After air drying for at least 24 h, sections were incubated in 0.025% cresyl violet (Merck, Darmstadt, Germany) and 0.05% acetic acid (VWR, Dorset, UK) at 56 °C for 30 min. Sections were then differentiated through a series of graded concentrations of industrial methylated spirits (IMS, VWR) and cleared in xylene (VWR) for at least 24 h before coverslipping with DPX (VWR), a xylene-based mountant.

### Immunohistochemical staining

To assess lysosomal storage and microglial activation in the forebrain and cerebellum, regularly spaced sections were selected, and immunohistochemically stained, using standard protocols^33^, for the markers LAMP1 (lysosomal membrane protein 1, lysosomes) and Iba1 (ionized calcium binding adaptor molecule 1, a calcium binding protein specific to microglia and macrophages), in addition to GFAP (glial fibrillary associated protein, astrocyte activation) in the cerebellum. Briefly, after quenching endogenous peroxidase activity in 1% H_2_O_2_ (Fisher Scientific) for 25 min, sections were rinsed, and to reduce non-specific protein binding were incubated with 15% normal serum (serum from the host species of the secondary antibody) in TBS-T (TBS containing 0.3% IBA/v Triton X-100) for 30 min. Primary antibody was applied in a 10% serum solution in TBS-T (Polyclonal goat anti-Iba1, 1:1000; polyclonal rabbit anti-LAMP1, 1:500, Abcam, Cambridge, UK; polyclonal rabbit anti-GFAP, 1:2000, Dako Ltd, Ely, UK), and left to incubate overnight at room temperature. After rinsing, sections were incubated with secondary antiserum (rabbit anti goat for Iba1, 1:1000; Vector; swine anti-rabbit for LAMP1 & GFAP; Dako, Cambridgeshire, UK) in TBS-T with 10% normal swine or rabbit serum, respectively, for 2 h. After rinsing with TBS, sections were incubated overnight with avidin–biotin-peroxidase complex (Vectastatin Elite ABC kit, Vector Laboratories, 1:1000). To visualize immunoreactivity, slides were incubated in a 0.05% solution of 3, 3′- diaminobenzidine tetrahydrochloride (DAB; Sigma, Dorset, UK) with 0.001% H_2_O_2_ solution in TBS for approximately 8 min. The reaction was stopped with ice-cold TBS. After rinsing with TBS, sections were mounted on Superfrost Plus microscope slides (ThermoFisher Scientific), and air dried overnight. Slides were then cleared with xylene, and coverslipped with DPX (VWR).

### Cortical thickness

To assess potential differences in cortical thickness, Nissl-stained sections were measured perpendicularly from the pial surface to white matter border, and this thickness was analyzed with *StereoInvestigator* software (MBF Bioscience Inc., Williston, VT, USA). These measurements were made in the primary somatosensory cortex, the rostral suprasylvian gyrus; this region was identified according to neuroanatomical landmarks^34^. Three sections containing this region were selected from each brain, and slides were blinded to age and treatment before analysis started. Ten measurements of cortical thickness were made per section, and these measurements were made in areas as far from gyrus curvature as possible to minimize variability. The mean regional cortical thickness was then calculated for each brain.

### Stereological counts of neuron number

Optical fractionator counts of the number of Nissl-stained DCN neurons were made using an appropriate grid size and dissector frame. Only neurons with a clearly identified morphology that fell within the dissector frame were counted. For all optical fractionator estimates, the mean coefficient of error of individual estimates was calculated according to the method of Gundersen and Jensen^35^ and was less than 0.08 in all these analyses. The number of Purkinje neurons was counted manually and expressed as the number per unit length within the same folium in each brain.

### Measurements of microglial area

The mean size of Iba1-stained microglia in the forebrain was obtained by measuring the cross-sectional area of at least 100 morphologically identified Iba1-stained microglia in each animal using *StereoInvestigator* software, and the mean microglial area determined ± standard area of the mean (SEM).

### Thresholding image analysis

The expression of GFAP, Iba1, and LAMP1 was measured in the cerebellum by semi-quantitative image thresholding analysis of each antigen. From three consecutive immunostained sections, 10 non-overlapping images were captured from each section for each of the areas examined. All images were captured at 40X objective by a live video camera (JVC, 3CCD, KY-F55B) mounted on Zeiss Axioplan universal microscope (Germany). The lamp intensity, video camera setup, and calibration were kept constant throughout the image capturing process. *Image-Pro Plus 5.0* software (MediaCybernetics, Chicago, IL, USA) was used to analyze the images with an appropriate threshold selected to distinguish the foreground immunostaining above the background. Macros were recorded to transfer the data to a spreadsheet for statistical analysis. The data obtained from the thresholding analysis was plotted graphically as a mean percentage area of immunoreactivity per treatment group ± SEM.

### Statistical analysis

All statistical analyses were performed blinded to genotype and age, which was only revealed once all morphological analyses were completed. A Kolmogorov–Smirnov test, a nonparametric test which assesses the empirical distribution functions of two samples, was applied to measured microglial cell body areas (GraphPad Prism, La Jolla, CA). A t-test was used to determine whether there was a statistically significant difference between genotype groups in cortical thickness of somatosensory cortex. Results were considered statistically significant if they produced a *p* value less than or equal to 0.05.

## Supplementary information

Supplementary Information.

## Data Availability

The data sets analyzed in this study are available from the corresponding author upon reasonable request.

## Abbreviations

CNS: Central nervous system
CSF: Cerebrospinal fluid
DCN: Deep cerebellar nuclei
ERT: Enzyme replacement therapy
GAG: Glycosaminoglycan
GFAP: Glial fibrillary associated protein
HS: Heparan sulfate
HS-NRE: Non-reducing end of HS
Iba1: Ionized calcium binding adaptor molecule 1
LAMP1: Lysosomal membrane protein 1
LSD: Lysosomal storage disorder
MPS: Mucopolysaccharidosis
NAGLU: Alpha-*N*-acetylglucosaminidase
SEM: Standard error of mean
TBS: Tris-buffered saline
