# Supplementary material for: Central nervous system pathology in preclinical MPS IIIB dogs reveals progressive changes in clinically relevant brain regions
Source: Sci Rep. 2020 Nov 23;10:20365. doi: 10.1038/s41598-020-77032-y (PMC7684310; doi:10.1038/s41598-020-77032-y)
Supplement: Supplementary file 1 — Supplementary Information. [file 41598_2020_77032_MOESM1_ESM.docx]

**Central nervous system pathology in preclinical MPS IIIB dogs reveals progressive changes in clinically relevant brain regions**

Martin T. Egeland^aǂ^, Marta M. Tarczyluk-Wells^bǂ^, Melissa M. Asmar^b^, Evan G. Adintori^c^, Roger Lawrence^c^, Elizabeth M. Snella^d^, Jackie K. Jens^d^, Brett E. Crawford^c^, Jill C.M. Wait^c^, Emma McCullagh^c^, Jason Pinkstaff^c^, Jonathan D. Cooper^a,b,*^, N. Matthew Ellinwood^d^

**Supplementary Material**

**Supplementary Figures 1 and 2**

^a^The Lundquist Institute at Harbor-UCLA Medical Center, and David Geffen School of Medicine, UCLA, Torrance, CA, USA

^b^King’s College London, Institute of Psychiatry, Psychology & Neuroscience, Maurice Wohl Clinical Neuroscience Institute, London, UK

^c^BioMarin Pharmaceutical Inc., Novato, CA, USA

^d^Iowa State University, Ames, IA, USA

^ǂ^These authors contributed equally to this work

**Supplemental Material**


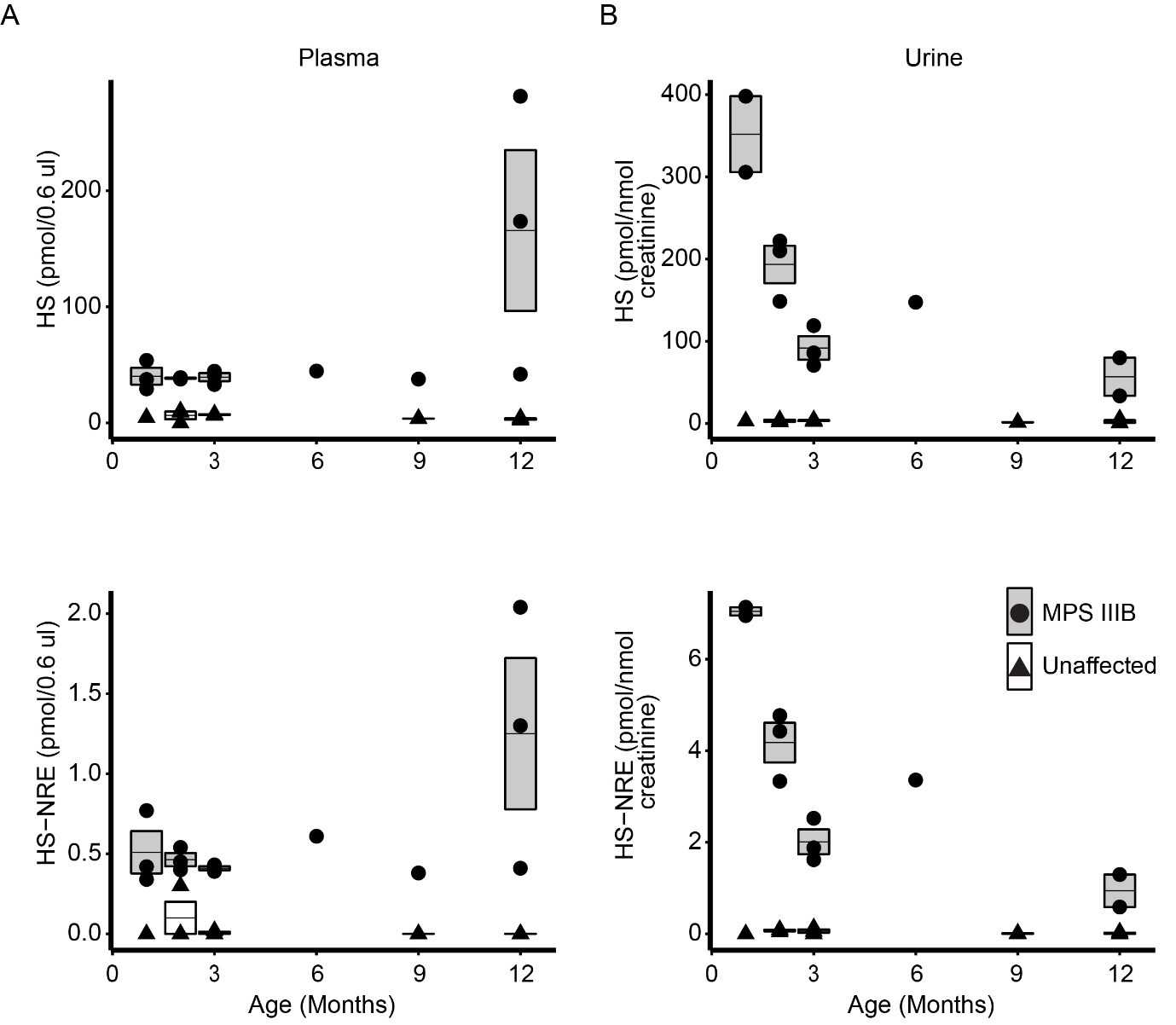


**Supplementary Figure 1.** Levels of heparan sulfate (HS) and the MPS IIIB-specific non-reducing end of heparan sulfate (HS-NRE) in plasma and urine of MPS IIIB and unaffected dogs. LCMS quantification of HS levels (top row) and HS-NRE (bottom row) in plasma **(A)** and urine **(B)** of MPS IIIB (circles, grey bars) and unaffected (squares, empty bars) dogs at different ages from 1 month to 12 months old. Levels of HS and HS-NRE in the urine were normalized to creatinine. Bars illustrate the mean (horizontal line) and standard deviation of data for time points at which samples from more than two dogs were analyzed. Data points with a horizontal line through them but no bar represent more than one sample was analyzed but the variability was extremely small. Data points with no horizontal line indicate a sample from a single animal was analyzed.

**Supplementary Figure 2.** Sulfation and acetylation pattern of heparan sulfate (HS) in brain (cortical grey matter) and cerebrospinal fluid (CSF) of MPS IIIB dogs. Cumulative percentage of total HS in the cortical grey matter **(A)** and CSF **(B)** of 1-, 3-, and 12-month-old MPS IIIB dogs that is 2-O sulfated (2S), 6-O sulfated (6S), N-sulfated (NS), and N-acetylated (NAc), as quantified by LCMS. N ≥ 3 at each time point.


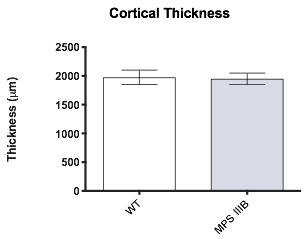


**Thickness (µm)**

**Unaffected**

**MPS IIIB**

**Supplementary Figure** **3**. Lack of atrophy in the MPSIIIB somatosensory cortex. Measurements of the thickness of the thickness of the rostral suprasylvian gyrus revealed no significant difference between unaffected and MPS IIIB dogs, even in the oldest cohort (>20 months).
